# Paradigm Shift Toward “One Health” Monitoring of *Culex*-Borne Arbovirus Circulation in France: The 2022 Inaugural Spotlight on West Nile and Usutu Viruses in Nouvelle-Aquitaine

**DOI:** 10.1093/ofid/ofaf243

**Published:** 2025-04-19

**Authors:** Gaëlle Gonzalez, Camille Victoire Migné, Alexandre Duvignaud, Sandra Martin-Latil, Clément Bigeard, Thierry Touzet, Albin Fontaine, Stephan Zientara, Xavier de Lamballerie, Denis Malvy

**Affiliations:** Anses, INRAE, Ecole Nationale Vétérinaire d'Alfort, UMR Virologie, Laboratoire de Santé Animale, Maisons-Alfort, France; Anses, INRAE, Ecole Nationale Vétérinaire d'Alfort, UMR Virologie, Laboratoire de Santé Animale, Maisons-Alfort, France; Department of Infectious Diseases and Tropical Medicine, CHU Bordeaux, Bordeaux, France; National Institute for Health and Medical Research (INSERM) UMR 1219, Research Institute for Sustainable Development (IRD) EMR 271, Bordeaux Population Health Research Centre, University of Bordeaux, Bordeaux, France; Anses, INRAE, Ecole Nationale Vétérinaire d'Alfort, UMR Virologie, Laboratoire de Santé Animale, Maisons-Alfort, France; Anses, INRAE, Ecole Nationale Vétérinaire d'Alfort, UMR Virologie, Laboratoire de Santé Animale, Maisons-Alfort, France; Department of Infectious Diseases and Tropical Medicine, CHU Bordeaux, Bordeaux, France; National Institute for Health and Medical Research (INSERM) UMR 1219, Research Institute for Sustainable Development (IRD) EMR 271, Bordeaux Population Health Research Centre, University of Bordeaux, Bordeaux, France; Direction Départementale de la Protection Des Populations (DDPP) de la Gironde; Direction Départementale de la Protection Des Populations (DDPP) de la Gironde; Unité des Virus Émergents (UVE: Aix-Marseille Univ, Università di Corsica, IRD 190, Inserm 1207, IRBA), Marseille, France; Institut de Recherche Biomédicale des Armées (IRBA), Unité de Virologie, Marseille, France; Anses, INRAE, Ecole Nationale Vétérinaire d'Alfort, UMR Virologie, Laboratoire de Santé Animale, Maisons-Alfort, France; Unité des Virus Émergents (UVE: Aix-Marseille Univ, Università di Corsica, IRD 190, Inserm 1207, IRBA), Marseille, France; Department of Infectious Diseases and Tropical Medicine, CHU Bordeaux, Bordeaux, France; National Institute for Health and Medical Research (INSERM) UMR 1219, Research Institute for Sustainable Development (IRD) EMR 271, Bordeaux Population Health Research Centre, University of Bordeaux, Bordeaux, France

**Keywords:** emergence, One Health, operational research, preparedness, West Nile virus

## Abstract

Global changes have profoundly altered the interactions between pathogens and their hosts, accelerating the emergence of infectious diseases. Monitoring vector-borne infectious diseases is therefore challenging and requires an upgrading of the detection system relying mainly nowadays on passive surveillance and reactive measures when a human case is diagnosed. West Nile virus (WNV) and Usutu virus are 2 zoonotic orthoflaviviruses, maintained between bird populations and mosquitoes, threatening public and veterinary health in Europe. In 2022, WNV unexpectedly emerged on the Atlantic coast of France in equids. Following this emergence, a consortium of national and local actors from the Nouvelle-Aquitaine region conducted crucial operational research, integrating environmental and animal data to make timely evidence-based and territorialized decisions to better assess the risk to human health. The proposal outlines the creation of a novel collaborative effort uniting experts from veterinary, human, and environmental health, as well as policy-makers. This partnership aims to establish a sustainable framework to address persistent knowledge gaps in our comprehension of arboviral disease emergence. By integrating diverse scientific disciplines with institutional decision-making processes, the initiative seeks to enhance our understanding of the complex factors contributing to the emergence and spread of arboviral diseases.

Several mosquito-borne viral diseases have emerged globally over recent decades, with both a significant extension in their circulation area and an increase in their caseload, putting an additional burden on human and veterinary public health services [1]. Keeping in mind the recent coronavirus disease 2019 (COVID-19) pandemic, the European scientific and public health community rushed to develop a high-level agenda to implement the concept of a holistic interconnected “One Health” approach. This challenging approach combines the human, animal, and environmental spheres in order to improve preparedness and response against the growing, (re)emerging zoonotic infectious diseases threat.

West Nile virus (WNV), responsible for some of the most notorious modern epidemics, and Usutu virus (USUV) are both mosquito-borne viruses from the *Orthoflavivirus* genus, formerly known as *Flavivirus*, within the *Flaviviridae* family. Native to Africa, WNV and USUV were introduced to Europe through import corridors driven by migratory birds [2, 3]. The viruses overlap in terms of geographic range, mosquito vector species, and amplifying resident avian hosts [4, 5]. Humans and horses are incidental dead-end hosts and do not develop viremia with a sufficient viral load to spread the disease. Infections can develop into severe and potentially fatal illness, affecting the central nervous system (ie, encephalitis, meningitis). WNV has a greater public health impact than USUV, but USUV geographic spread [6] and potential to induce neurological disease [7–9] are likewise a cause for concern. WNV and USUV are both classified into multiple genetic lineages based on their genetic diversity. The current classification proposes up to 9 and 5 distinct genetic lineages for WNV and USUV, respectively [10]. Lineages 1 and 2 are actively circulating in Europe, causing significant outbreaks and severe neurological diseases in humans, horses, and birds. The year 2018 was a first turning point in the epidemiology of both viruses. WNV lineage 2 infections exceeded the cumulative number of all previously reported infections [11]. Since 2018, USUV has spread through Europe, causing massive mortalities in wild/captive birds (ie, 46 French counties reported cases in 2018) and sporadic but nonetheless worrisome human cases [9]. A key milestone for WNV is the year 2022, characterized by the resurgence of WNV lineage 1 in Italy, which was associated with an increased rate of human fatalities [12, 13]. Both USUV and WNV are now circulating in endemic cycles in Europe, with viral maintenance from season to season through mosquito overwintering [14] and/or in resident bird species [15]. Reintroductions by migratory birds cannot be excluded. Most infections in these incidental human and equine hosts are asymptomatic or mild and thus go unreported, except when an active surveillance system is in place. France has a syndromic surveillance system, which limits virus detection to symptomatic cases [16–21].

Recently, France has launched national and local surveillance initiatives to respond rapidly and effectively to threatening emerging diseases. However, important gaps remain to facilitate the dissemination of surveillance data between national and local levels. Important efforts are required to facilitate early implementation of mitigation measures by local effectors in order to efficiently protect environmental, animal, and human health. Hence, strong coordination is necessary to shift from being reactive to being proactive in our response.

Herein, we aim to illustrate how the Nouvelle-Aquitaine region moved forward from a concept to an operational strategy in the context of the recent emergence of West Nile and Usutu viruses in this French region. We formed a consortium of academic, civil society, and governmental actors with the goal to intervene at the interface between human–animal–environmental sciences and conduct epidemiological investigation for better assessment of viral risk, such as One Health monitoring primarily aimed at helping policy-makers to make timely evidence-based and territorialized decisions to limit and anticipate potential consequences on human health from the spread of these 2 viruses. The knowledge gained through this regional experience may contribute to reshaping surveillance and preparedness policies, particularly through the integration of operational applied research as a key component for the prediction and early detection of arboviral emergence and circulation in France and Europe.

##  

### French Experience Dealing With Mosquito-Borne Zoonotic Pathogens

In recent decades, mainland France experienced numerous episodes of intense mosquito-borne orthoflavivirus circulation. This includes the reemergence of WNV, an avian virus transmitted by the indigenous *Culex* mosquito around the Mediterranean arc and, more recently, the establishment of WNV in the Southwestern part of the country (Nouvelle-Aquitaine), where it co-circulates with USUV. Additionally, with the establishment of *Aedes albopictus,* the number of autochthonous Dengue cases reported each summer has increased sharply [22], correlating with multiple viral introduction by infected travelers returning from French overseas endemic areas—mainly the Caribbean area [23]. Episodes of autochtonous chikungunya and Zika virus transmission have also been reported in recent years [24].

These 2 remarkable events could pave the way to seasonal outbreaks in areas where the climate is most suitable. To date, vector-borne disease control mainly relies on passive surveillance and reactive measures when a human case is detected. Within the One Health approach, the conventional response is characterized by limited yet proactive operational research efforts that promote both intrasector and intersector collaboration. Nonetheless, in the context of this reactive strategy, early suspicion, diagnostic confirmation, and case notification are of paramount importance to efficiently prevent and circumscribe epidemic clusters. The French health system surveillance is well defined, and the roles of different actors are clearly delineated. Indeed, at the national level, human surveillance is coordinated by the Direction Générale de la Santé and the human National Reference Center for arboviruses while veterinary surveillance is coordinated by the Direction Générale de l’Alimentation and the veterinarian National Reference Laboratory for WNV. This multidisciplinary passive surveillance system, established in 2001, is a cornerstone of France's strategy for the detection of WNV circulation in metropolitan areas. By assessing human, equine, and avian passive monitoring, it delivers real-time data, which are crucial for enacting targeted control and prevention measures. Surveillance efforts prioritize regions with a history of WNV activity, with heightened focus on the Mediterranean basin in Southern France and, since 2023, the Nouvelle-Aquitaine region in the southwest, where the virus is most prevalent. Each year, these areas are subject to campaigns for equine veterinarians, French Biodiversity Office collectors/discoverers, and frontline emergency physicians to detect the first cases of infections caused by *Culex*-borne *Orthoflavivirus*. When a detection in humans occurs, authorities implement a robust communication and response protocol to mitigate public health risks. Reports are immediately relayed to key stakeholders, including public health officials, veterinary services, and environmental health departments. Notifications are systematically sent to regional health agencies (Agences Régionales de Santé [ARS]) and national bodies like Santé Publique France and the Direction Générale de l’Alimentation (DGAL). Authorities quickly assess the geographic spread, the number of infected specimens, and environmental factors contributing to virus transmission, allowing for precise and effective action. In addition, France employs systematic viral screening of all blood and organ donors as soon as a human WNV case is diagnosed and reported. Genomic surveillance is expanded to encompass asymptomatic individuals in high-risk areas and local blood donors, enabling authorities to track the virus's silent circulation and preempt broader outbreaks. This vigilant and adaptive framework demonstrates France's commitment to safeguarding public health and interagency collaboration to counter the threat of WNV effectively. Despite the considerable amount of accumulated knowledge that is being produced each year, no formal anticipation and preparedness scenario has been established to mitigate future outbreaks and control the (re)emergence and dissemination of zoonotic pathogens. However, there is a common consensus that the emergence of vector-borne infectious diseases is the result of an interplay of factors at the interface of humans, animals, environmental ecosystems, vectors, and viruses that need to be studied following the above One Health approach, with a strong intersectoral integration at both the national and local levels. All the above elements argue for reinforced data-sharing and common action between the different players of the health system, that is, human, animal, and environmental systems.

### Breaking Silos in the Context of Emerging West Nile and Usutu Viruses in the Nouvelle-Aquitaine Region, Southwestern France

In 2022, France faced the unexpected emergence of WNV in the Southwestern part of the country, more specifically on the Atlantic coast in Gironde county (Nouvelle-Aquitaine region), and immediately afterward the biggest forest megafire that has ever been recorded in the area. Although our findings were at that time weak signals limited to a certain number of proven equine infection cases, the unexpected emergence of WNV has completely upset our certainties about the circulation of the virus being associated with the Mediterranean basin (Figure 1). The same year, an autochthonous human case of USUV infection was reported in Les Landes county, part of the same administrative region [25]. In response to these events, One Health integrated operational research initiatives were developed to characterize the emergence and circulation of these viruses in the Nouvelle-Aquitaine socio-ecosystem and to gain a better understanding of the multiple potential factors (eg, climatic and ecological changes, migratory bird routes, host and mosquito vectors’ abundance and dynamics) that embraced the emergence of WNV. A research consortium, bringing together regional and national partners, each an expert in their own field, has undertaken to produce integrated scientific data in the human, veterinary, and entomological sectors. In order to monitor the spread of WNV and USUV in Gironde county, we conducted studies to assess the seroprevalence rate in horses and the prevalence of viral genomes in dead birds and mosquitoes before the 2023 transmission season (Figure 2). The equine seroprevalence study showed a heterogeneous distribution of specific anti-WNV immunoglobulin G antibodies over the territory [26], with an average rate of 6.4%, close to the rate estimated in equids after the 2000 outbreak that occurred in the historical circulation area of Camargue in the Southeastern Mediterranean part of the country [27]. The consortium also tested an innovative, low-cost, easy-to-implement, and high-yield technology developed by the Institut de Recherche Biomédicale des Armées for detecting WNV and USUV viral genome in mosquito vectors, Molecular Xenomonitoring (MX) [28]. Usually, arbovirus surveillance deployed in Europe involves extensive mosquito collection, often amassing thousands to hundreds of thousands of specimens over a season. Mosquitoes are then pooled (50 to 200 specimens per pool) based on species, collection date, and location before processing to viral extraction and genomic detection. The minimum infection rates reported for WNV surveillance show considerable variation, from 2% [29–32] to 10% [29, 30]. MX succeeded in detecting WNV and USUV enzootic transmissions in Nouvelle-Aquitaine with a high rate of detection in trapped mosquito excreta (75% of samples) in late July 2023. This coincided with the confirmation of the first human WNV case in the region and occurred a few days before the first equine case was reported in Gironde county on August 4 [28]. The unrivalled performance of this breakthrough technology puts entomological surveys back at the heart of public health decision-making tools. The feedback from these investigations can inform health authorities' decisions to expand geographically and strengthen screening for viral genomic material in products of human origin (blood and tissue/organ graft) from asymptomatic donors, in order to avoid secondary transmission to vulnerable and immunocompromised patients. Besides, there are many other potential applications for other vector-borne diseases of importance to human (eg, autochthonous Dengue fever in mainland France) and animal health (eg, epizootic hemorrhagic fever).

**Figure 1.**
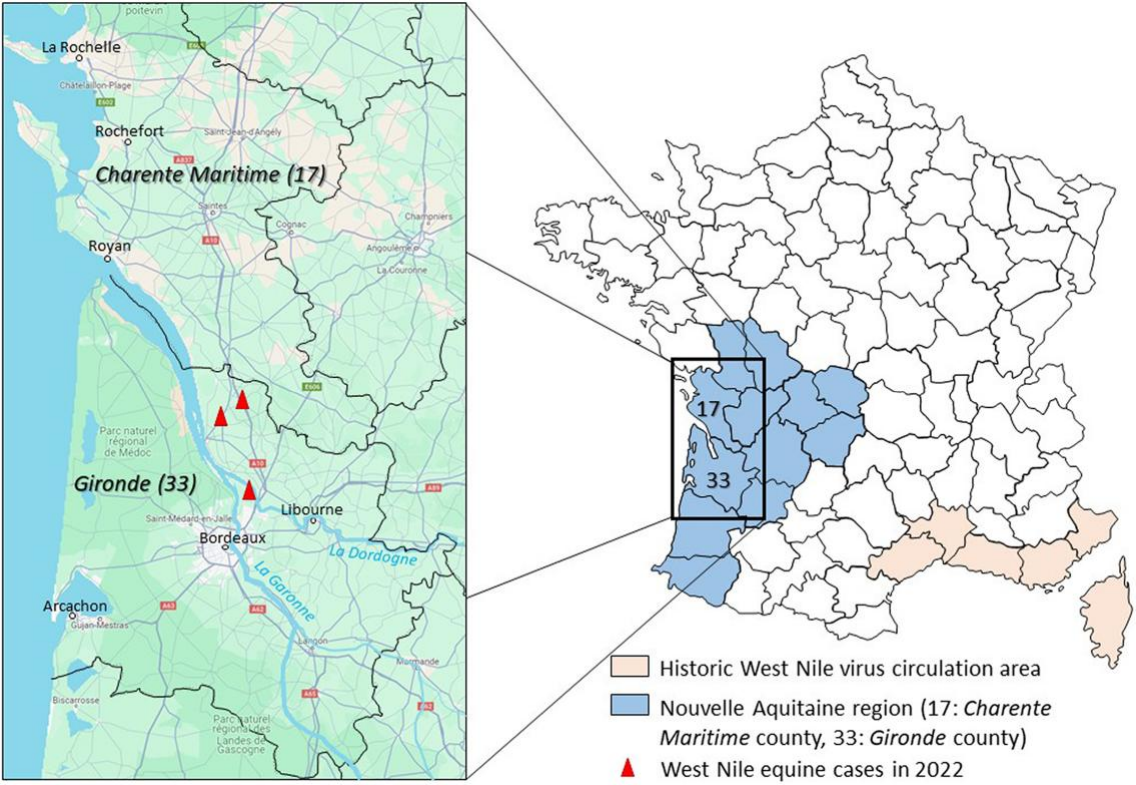
Nouvelle-Aquitaine region, the novel zone of emergence of West Nile virus in France since 2022. © Google, Imagery ©2025 TerraMetrics.

**Figure 2.**
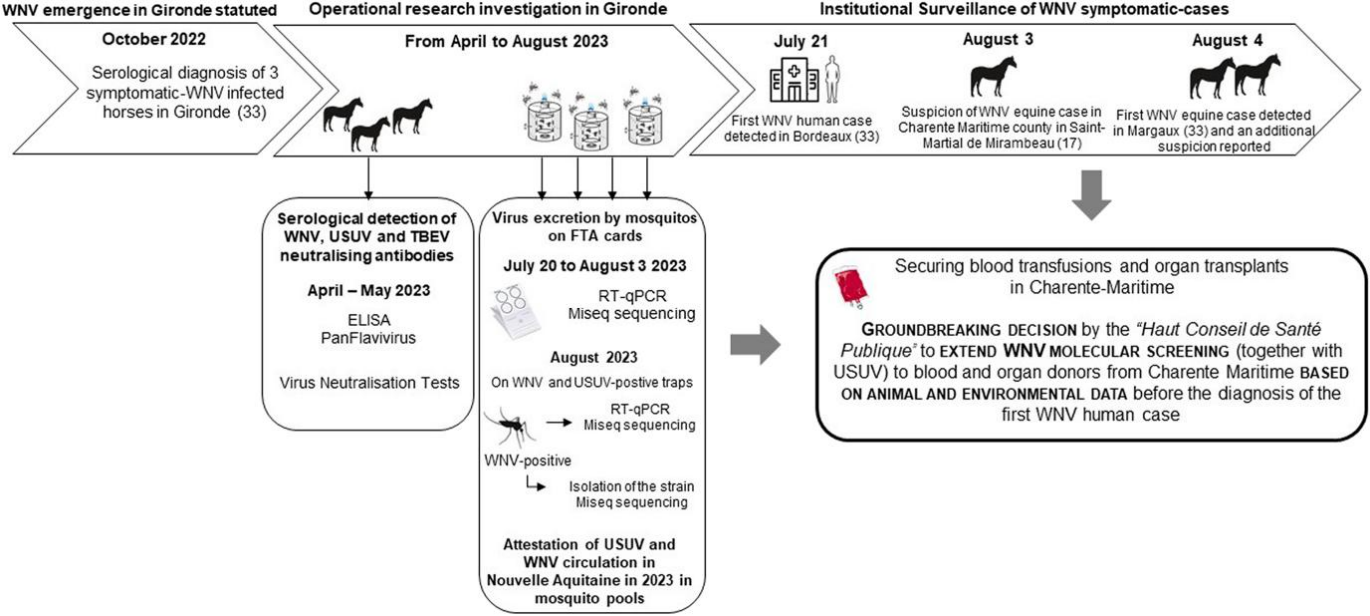
Timeline and sampling scheme of the operational research investigations conducted in Gironde. Abbreviations: ELISA, enzyme-linked immunosorbent assay; RT-PCR, reverse transcription quantitative polymerase chain reaction; TBEV, Tick-Borne Encephalitis virus; USUV, Usutu virus; WNV, West Nile virus.

The start of the 2023 transmission season was marked by the concomitant diagnosis in Gironde at the end of July of the first human cases of WNV infection, located in the Bordeaux metropolitan area (including the city of Bordeaux itself), as well as the first equine case of the year in the municipality of Margaux (Medoc area), followed in early August by suspicion of an equine case in the south of bordering Charente-Maritime county (municipality of Saint Martial de Mirambeau) (Figure 2).

Taken together, our early operational research findings and these surveillance data were instrumental in the Haut Conseil de Santé Publique’s unprecedented recommendation to extend WNV molecular screening (together with USUV) to blood donors from Charente-Maritime, where no human case had yet been detected. This groundbreaking decision was the first taken by French national human health authorities based on nonhuman environmental and veterinary data. This constitutes a turning point in *Culex*-borne zoonosis monitoring and control policies in France, previously founded on the occurrence of the first human case in a given area of the country.

## PERSPECTIVES

Here, we illustrate how a consortium of national and regional actors applied the “One Health” approach, which is universally recognized as the cornerstone for efficient prevention and control of emerging and re-emerging zoonotic infectious diseases. The data gathered by our consortium have proved a crucial complement to institutional syndromic surveillance. The approach demonstrates how appropriate investigation of signals considered to be weak provides crucial information to address the occurrence and burden of sequential cluster emergence. The Haut Conseil de la Santé Publique implemented enhanced control measures to protect public health in response to several critical factors indicating increased WNV activity such as (1) high WNV seroprevalence in equids in Gironde, just 1 year after the virus's initial emergence in the region, (2) detection of both WNV and USUV at high infection rates in mosquito pools, (3) confirmation of the first human WNV case in the region, and (4) a suspected equine WNV case in Charente-Maritime. These findings collectively signaled an expanding and intensifying arboviral circulation in the area. Indeed, operational research plays a pivotal role in the strengthening of the French *Culex*-borne arboviral disease monitoring system through production of highly relevant contextualized evidence consisting of WNV seroprevalence rates in equids and/or birds, which is associated with detection of the virus in mosquito pools, which is instrumental for designing efficient risk mitigation strategies. Although WNV and USUV are considered endemic in Europe, there is nowadays no effective way of anticipating the emergence of these viruses in new territories. An important gap to be filled is the understanding of the factors underlying viral emergence in a given area.

Even if the operational research data produced by our consortium were patchy, they suggested the active circulation of WNV and USUV in a vast socio-ecosystem stretching from the Bordeaux metropolis to both banks of the Gironde estuary. This large area of concern could extend upstream into the marshland areas along the Garonne and Dordogne rivers and their tributaries, as well as to the bordering counties of Nouvelle-Aquitaine where local conditions are also suitable for the establishment of these 2 pathogens but where their circulation is still to be investigated. This is important given the immediate proximity of these potential traffic areas to the densely populated and summer tourist urban areas of Arcachon, Libourne, Royan, Rochefort, and La Rochelle (Figure 1).

The focus will be first placed on sustaining and extending the interdisciplinary One Health approach needed to mitigate *Culex*-borne arbovirus-related burden in the context of global change [33, 34]. In order to strengthen national surveillance, optimize the effectiveness of operational research, and anticipate outbreaks of West Nile fever and West Nile neuroinvasive disease, it is essential to involve the public [35, 36]. Science generated by citizen involvement associated with digital approaches (internet, mobile phones) and social networks is opening up a new dimension in applied research into the ecology of vectors and their hosts (abundance, location, mobility, etc.), virus–host–environment interactions, and virus transmission zones at different scales (local, territorial, etc.). In the context of the global environmental and climate challenges and meeting the 2030 Sustainable Development Goals, nature-based solutions (NBS) are recognized as key assets in dealing with climate change consequences. Urban greening has demonstrated its benefits by enhancing biodiversity and promoting healthy ecosystems in an increasingly urbanized world, but it may also be profitable for emerging vector-borne diseases (EVBDs) [37–39]. Risk assessment regarding the impact of such solutions on emerging pathogens such as WNV needs to be conducted looking at the vectors' distribution, the avian host's abundance and behavior, and the interaction of vectors–hosts–viruses in the modified urbanized environment. Cutting edge digital technologies teamed with citizens and NBS will allow accurate monitoring of EVBDs, which is essential for local, national, and international preparedness against arboviral threats.

Moreover, the identification of early determinants of viral emergence (ie, introduction, amplification, and species barrier jump) in the territory are crucial. In particular, this will involve studying the climatic, ecological, behavioral, and sociological factors that trigger the expression of this emergence, including the impact of critical environmental events and land use planning. We aim to convert these investigations into recommendations to policy-makers in preparing, anticipating, and responding to outbreaks.

To achieve this goal, there is a need to consolidate and expand the so far informal network of regional and national stakeholders set up in 2023 and to put it in the French national One Health agenda in the context of the future interministerial plan to tackle human and animal arboviral diseases. The initiative presented here narrates the development of an original partnership between veterinary, human, environmental sciences and institutional decision-makers in the realm of sustainability with the goal of fielding remaining gaps in our understanding of arboviral emergence.

Such operational research actions backed with institutional surveillance will accelerate the acquisition of knowledge and inform the design of preparedness programs.
